# Collagen VI sustains cell stemness and chemotherapy resistance in glioblastoma

**DOI:** 10.1007/s00018-023-04887-5

**Published:** 2023-07-28

**Authors:** Matilde Cescon, Elena Rampazzo, Silvia Bresolin, Francesco Da Ros, Lorenzo Manfreda, Alice Cani, Alessandro Della Puppa, Paola Braghetta, Paolo Bonaldo, Luca Persano

**Affiliations:** 1 Department of Molecular Medicine, University of Padova, Via Ugo Bassi 58/B, 35131 Padua, Italy; 2 Department of Women and Children’s Health, University of Padova, Via Giustiniani 3, 35127 Padua, Italy; 3 Istituto di Ricerca Pediatrica-Città della Speranza, Corso Stati Uniti 4, 35128 Padua, Italy; 4 Department of Neuroscience, Psychology, Pharmacology and Child Health, Neurosurgery Clinic, Academic Neurosurgery, Careggi University Hospital and University of Florence, Largo Palagi 1, 50139 Florence, Italy

**Keywords:** Glioblastoma, Extracellular matrix, Collagen VI, Cancer stem cells, Therapy resistance, DNA repair

## Abstract

Microenvironmental factors are known fundamental regulators of the phenotype and aggressiveness of glioblastoma (GBM), the most lethal brain tumor, characterized by fast progression and marked resistance to treatments. In this context, the extracellular matrix (ECM) is known to heavily influence the behavior of cancer cells from several origins, contributing to stem cell niches, influencing tumor invasiveness and response to chemotherapy, mediating survival signaling cascades, and modulating inflammatory cell recruitment. Here, we show that collagen VI (COL6), an ECM protein widely expressed in both normal and pathological tissues, has a distinctive distribution within the GBM mass, strongly correlated with the most aggressive and phenotypically immature cells. Our data demonstrate that COL6 sustains the stem-like properties of GBM cells and supports the maintenance of an aggressive transcriptional program promoting cancer cell proliferation and survival. In particular, we identified a specific subset of COL6-transcriptionally co-regulated genes, required for the response of cells to replicative stress and DNA damage, supporting the concept that COL6 is an essential stimulus for the activation of GBM cell response and resistance to chemotherapy, through the ATM/ATR axis. Altogether, these findings indicate that COL6 plays a pivotal role in GBM tumor biology, exerting a pleiotropic action across different GBM hallmarks, including phenotypic identity and gene transcription, as well as response to treatments, thus providing valuable information for the understanding of the complex microenvironmental cues underlying GBM malignancy.

**Supplementary Information:**

The online version contains supplementary material available at 10.1007/s00018-023-04887-5.

## Introduction

Glioblastoma multiforme (GBM) is the most common malignant brain tumor, and one of the most devastating human cancers, showing resistance to multimodal treatments. GBM patients display a median survival of 16–18 months after diagnosis and a 3-year survival rate of less than 15% [1, 2]. GBM contains a subset of cancer stem cells, known as glioma stem cells (GSCs), which are considered responsible for therapy resistance and tumor relapse [3, 4]. We previously reported the fundamental role played by the GBM hypoxic microenvironment in sustaining a GSC phenotype and driving a concomitant intrinsic resistance to chemotherapy [5]. In particular, we demonstrated that GBM cells distribute along a core-to-periphery gradient within the tumor mass, in parallel with variations in phenotype, proliferation, tumorigenic potential, and response to treatments, depending on hypoxia and, in particular, on HIF-1α activation [5–8].

Within tumor microenvironment, the extracellular matrix (ECM) plays multiple key roles by shaping stem cell niches [9], modulating growth factors availability [10], mediating specific ECM-dependent pro-survival signaling cascades [11], influencing tumor invasiveness [12] and response to chemotherapy [13], and even providing chemoattractant cues for inflammatory cells [14]. A number of studies demonstrated that collagen VI (COL6), an ECM protein widely distributed in several tissues, including brain vessels, and in close association with basement membranes [15, 16], is involved in tumor growth [17]. COL6 is made of three different subunits, α1(VI), α2(VI), and α3(VI) chains, characterized by a relatively short collagenous region and by large globular domains at the N- and C-terminal ends [18]. Once secreted, the protein generates a distinctive network of extracellular beaded microfilaments interacting with several other ECM components and providing mechanical, pro-survival, and antiapoptotic stimuli to cells [19–24]. COL6 is highly expressed in a variety of human tumors, including breast, pancreatic and lung cancers, and in metastatic tissues [17, 25–28], where it modulates cell proliferation, angiogenesis, and inflammation [29–32]. Notably, a cleavage product of α3(VI), named endotrophin (ETP), was shown to promote tumor progression, angiogenesis, fibrosis, and macrophage recruitment in a mammary tumor mouse model [31], while its inhibition increases tumor sensitivity to cisplatin [33].

Increased expression of COL6 was reported in GBM and high-grade gliomas, when compared to lower-grade astrocytoma and normal glia [26, 34], also correlating with a poor clinical outcome [35]. Despite these findings, the cellular mechanisms triggered by COL6 were never investigated in depth in this type of tumor. Here, we first corroborated the high expression of *COL6A1*, *COL6A2,* and *COL6A3* within a subset of ECM-related genes in different GBM patients’ datasets, compared to lower-grade tumors and normal brain tissues. Moreover, we identified a gradient of COL6 deposition within human GBM samples, showing higher levels in the tumor core and then decreasing toward the peripheral regions of the GBM mass. In vitro experiments demonstrated that modulation of COL6 levels heavily affects the pro-differentiative potential of GSCs, de facto preventing, or enhancing their serum-induced differentiation upon COL6 supplementation or suppression, respectively. Notably, gene expression profiling of COL6-silenced cells revealed that COL6 sustains a pro-cancerous transcriptional program, and its downregulation is highly correlated to the expression of a subset of genes involved in DNA replication and repair in GBM cells, significantly affecting their response to DNA damage and stress, eventually sensitizing them to chemotherapy. Collectively, these data demonstrate that COL6 is dramatically involved in the maintenance of a stem-like phenotype in GBM cells, with a clear impact on their response to treatments by sustaining their DNA repair capacity.

## Materials and methods

### Neurosurgical specimen collection and isolation of primary cultures.

Tissue specimens and primary cultures were isolated from GBM tumors at surgery. All tissues were acquired following the tenets of the Declaration of Helsinki. A differential sampling of tumor biopsies from either the GBM core, the more peripheral regions of the mass, and an intermediate transition layer between them was obtained by a T1-weighted MRI-based intraoperative neuro-navigation and a pre-identified image-guided collection of biopsies as previously described [8, 36]. General clinical features of patients from which GBM specimens and primary cultures used in this study were obtained are listed in Suppl. Table S1.

Primary GBM cultures were isolated and cultured as described previously [5, 36]. Briefly, GBM samples were enzymatically and mechanically dissociated into single cell suspensions. Cells were then placed on fibronectin-coated plates and grown as monolayers in DMEM/F12 (Biowest, Nuaillé, France) supplemented with 10% BIT9500 (Stem Cell Technologies, Vancouver, Canada), 20 ng/ml basic fibroblast growth factor (bFGF), and 20 ng/ml epidermal growth factor (EGF; both from Cell Guidance Systems Ltd., Cambridge, UK). GBM cells were maintained in an atmosphere of 2% oxygen, 5% carbon dioxide and balanced nitrogen in a H35 hypoxic cabinet (Don Whitley Scientific Ltd., Shipley, UK), to mimic the hypoxic conditions of GBM microenvironment, or exposed to environmental oxygen, according to specific experimental needs.

To induce differentiation, culture media was supplemented for 72 h with 10% fetal bovine serum (FBS; Thermo Fisher Scientific, Waltham, MA). When indicated, soluble native COL6 (snCOL6; 1 μg/ml any other day, purified as already described [37, 38]), or the pepsin-resistant triple helical fragment of COL6 (pepCOL6; 250 ng/ml, purified as described in [39, 40]) was added to the culture medium, or culture dishes were coated with purified native COL6 (cnCOL6; 5 μg/cm^2^) added as an adhesion substrate.

Brightfield images of cultured cells were acquired with a Nikon TS100 inverted microscope (Nikon, Melville, NY).

In some experiments, GBM cell growth was monitored along a 48 h time span by Trypan Blue (Sigma–Aldrich, St. Louis, MO) exclusion daily counts.

### Immunofluorescence and confocal microscopy

Immunofluorescence on formalin-fixed paraffin-embedded GBM tumor sections (5–7 μm thick) was performed according to the standard procedures. Briefly, slides were deparaffinized through xylene/ethanol rehydration, followed by phosphate-buffered saline (PBS) washing. Antigen unmasking was performed with a heat-mediated antigen retrieval step in citrate buffer (10 mM citric acid, pH 6.0), using a steamer brought to 100 °C for 20 min, followed by hyaluronidase treatment (500 U/ml in 150 mM NaCl, 20 mM Na acetate, pH 6.0) at room temperature for 30 min. Slides were washed in PBS, permeabilized in 0.2% Triton X-100 (Sigma-Aldrich, St. Louis, MO) in Tris-buffered saline (TBS) for 10 min, washed again and blocked in 1% bovine serum albumin (BSA; Sigma-Aldrich, St. Louis, MO) in TBS for 1 h. Primary antibodies against ColVI α3 (1:100, kindly supplied by Dr. Raimund Wagener, University of Cologne, Germany [41];), nestin (1:200; Millipore, Burlinghton, MA), Ki67 (1:100; Dako, Glostrup, Denmark), CD34 (1:50; Novocastra, Newcastle upon Tyne, UK), collagen VII (1:200; Sigma-Aldrich, St. Louis, MO), and collagen IV (1:250; Sigma-Aldrich, St. Louis, MO) were incubated overnight at 4 °C. After washing in PBS, samples were incubated with fluorescent secondary antibodies anti-rabbit IRIS 5.5 (1:200; Cyanine Technologies, Turin, IT), anti-mouse Cy3 (1:500), anti-mouse Cy2 (1:300), and anti-mouse Cy3 (1:500) (all from Jackson Immunoresearch, Ely, UK). Hoechst 33528 (Sigma-Aldrich, St. Louis, MO) was used for staining nuclei. Images were acquired with a Leica TCS SP5 confocal microscope (Leica Microsystems, Wetzlar, Germany). The positive area for COL6 staining was quantified on randomly chosen fields by ImageJ.

Immunofluorescence on GBM cultures was performed in 4-well chamber slides (BD Bioscience, Franklin Lakes, NJ) in the absence or presence of FBS and/or nCOL6. After 72 h, cells were fixed in cold 4% formaldehyde, and then washed and stored at 4 °C in PBS prior to analysis. Primary antibodies against Nestin (1:200; Merck-Millipore, Darmstadt, Germany), βIII-tubulin (1:500; Biolegend Inc., San Diego, CA), GFAP (1:500; Agilent Technologies, Santa Clara, CA), phospho-Vimentin (1:100; MBL International, Woburn, MA), Nanog (1:400; BD Bioscience, Franklin Lakes, NJ), and S100 (1:400; Agilent Technologies, Santa Clara, CA) were incubated according to manufacturer’s instructions. Cells were then washed and incubated with secondary antibodies conjugated to Alexa dyes (1:1000; Thermo Fisher Scientific, Waltham, MA). Nuclei were counterstained with DAPI (1 μg/ml; Sigma-Aldrich, St. Louis, MO). The stained samples were visualized with an Axio Imager M1 epifluorescence microscope (Zeiss, Jena, Germany).

### Reverse transcription and quantitative real-time RT-PCR

Total RNA (1–2 μg) was extracted from GBM cells using QIAzol reagent (Qiagen, Hilden, Germany) according to manufacturer’s instructions. RNA was reverse-transcribed using the SuperScript™ First-Strand Synthesis System (Thermo Fisher Scientific, Waltham, MA). Quantitative RT-PCR reactions were run in duplicate using Platinum SYBR Green Q-PCR Super Mix (Thermo Fisher Scientific, Waltham, MA). Fluorescent emission was recorded in real-time (Sequence Detection System 7900HT, Applied Biosystems, Foster City, CA). Primers used are listed in Suppl. Table S2. Primer specificity was assessed by alignment to Human BLAST Search (http://genome.ucsc.edu) and confirmed for every PCR run by dissociation curve analysis. Expression values were normalized to *GUSB* according to the ΔΔCt method.

### Western blotting

Equal amounts of proteins extracted from GBM cells (generally 10 µg) were resolved by SDS-PAGE gels (NuPage; Thermo Fisher Scientific, Waltham, MA) and transferred to polyvinylidene difluoride (PVDF) Immobilon-p membrane (Merck-Millipore, Darmstadt, Germany). Membranes were saturated with 2% I-block™ (Thermo Fisher Scientific, Waltham, MA) or 3% BSA (Sigma-Aldrich, St. Louis, MO) for at least 1 h at room temperature and then incubated overnight at 4 °C under constant shaking with antibodies against ColVI α1 (1:1000; Santa Cruz Biotechnology, Santa Cruz, CA), γH2aX (1:1000; Santa Cruz Biotechnology, Santa Cruz, CA), phospho-Chk1 (Ser345; 1:1000; Cell Signaling Technology, Danvers, MA), phospho-Chk2 (Ser33/35; 1:1000; Cell Signaling Technology, Danvers, MA), and β-actin (1:25,000; Sigma-Aldrich, St. Louis, MO). Membranes were then incubated with peroxidase-conjugated secondary antibodies (Perkin Elmer or Bethyl Laboratories, Waltham, MA) and visualized using ECL Select (Cytiva, Marlborough, MA). Images were acquired by using the iBright FL1500 Imaging System (Thermo Fisher Scientific, Waltham, MA).

### Limiting dilution assay

To assess GBM cell self-renewal, cells were seeded in 6 well plates, in the presence or absence of 10% FBS and/or 1 μg/ml of snCOL6 (added to the medium any other day) for 72 h prior to be re-plated in serial dilutions ranging from 0 to 100 cells/well in ultralow attachment 96-well plates through a MoFlo XDP cell sorter (Beckman Coulter, Brea, CA). Cells were cultured for two additional weeks and the percentage of wells displaying no sphere formation was measured, graphed, and used for initiating cell frequency estimation.

### Transfection of GBM cells and gene silencing

GBM cells were transfected using a protocol for transient transfection of adherent cells with the TransIT®-X2 Transfection Reagent (Mirus Bio LLC, Madison, WI). A Negative Control siRNA (siNEG) and two different Stealth siRNAs against human *COL6A1* transcript were purchased from Thermo Fisher Scientific and used at 50 pmol per transfection reaction (siCOL6A1#1: HSS102131 and siCOL6A1#2: HSS102132). Efficiency of gene silencing was verified by western blotting after 48 h from transfection. In some experiments, 24 h after transfection cells were exposed to FBS and/or snCOL6 as described above.

### Gene expression profiling and data analysis

For microarray experiments, in vitro transcription, hybridization, and biotin labeling of RNA were performed according to the WT GeneChip Clariom™ S assay (Affymetrix, Santa Clara, CA). Microarray data (CEL files) were generated using default Affymetrix microarray analysis parameters (Command Console Suite Software). CEL files were normalized using the robust multiarray averaging expression measure of the Affy-R package (www.bioconductor.org). Differentially expressed genes between siNEG and siCOL6A1#1 or siCOL6A1#2 transfected GBM cells (HuTuP82) were identified using the Significance Analysis of Microarray (SAM) algorithm coded in the samr R package [42]. In SAM, we estimated the percentage of false-positive predictions (i.e., False Discovery Rate, FDR) with 100 permutations. Genes with an FDR < 0.05 were considered significant. Expression data were deposited into the Gene Expression Omnibus (GEO) database under Series Accession Number GSE226700 and are accessible without restrictions. Clustering analysis was generated with R software using Euclidean distance as a distance measure between genes and Ward.D method. Heatmap in Suppl. Fig. S1 was generated by the Morpheus bioinformatic tool (https://software.broadinstitute.org/morpheus/).

Enrichment analyses were performed on common differentially expressed genes between the two used COL6A1 siRNAs by applying over representation analysis in the C2cp (common pathways) gene sets. The most significant enrichments (FDR *q* value < 0.05) are reported.

Identification of co-expressed gene modules within sample types (siNEG vs. siCOL6A1-transfected cells) was performed through the CEMiTool R package [43] according to Pearson correlation statistics. Modules were identified and clustered by a dissimilarity threshold of 0.8 and a *p* < 0.1. GSEA was performed to measure the relative enrichment of identified gene modules into sample categories. Over representation analysis was performed on module M6, which contains the *COL6A1* transcript.

Kaplan–Meyer survival analysis was achieved by subgrouping patients into quartiles according to the sum of the mean-centered log_2_ expression values of the identified COL6 Highly Correlated Genes (HCG; *n* = 10).

### DNA gel electrophoresis

GBM cells were transfected with siCOL6A1 or siNEG using RNAiMAX reagent (Thermo Fisher Scientific, Waltham, MA) and, after 24 h, treated with Temozolomide (TMZ; 100–500 μM) or matched concentrations of DMSO for additional 24 h. DNA was extracted from GBM cells using QIAamp DNA mini kit (Qiagen, Hilden, Germany). Isolated DNA (1 μg) was then resolved in 1% agarose gel containing GelRed Nucleic Acid Stain (Biotium, Hayward, CA) in Tris–borate-EDTA (TBE) at 50 V for 3 h. Images were acquired by using the iBright FL1500 Imaging System (Thermo Fisher Scientific, Waltham, MA).

### Statistical analyses

Graphs and associated statistical analyses were generated using GraphPad Prism 8.0.1 (GraphPad, La Jolla, CA). All data in bar graphs are presented as mean ± standard error of the mean (SEM). Statistical significance was measured by one-way ANOVA (for more than two comparisons) and Student’s *t* test (comparison of two groups). For all graphs, asterisks over bars indicate a significant difference with the specific control group. Asterisks over brackets display a significant difference between indicated samples.

## Results

### Collagen VI is overexpressed in GBM

**Fig. 1:**
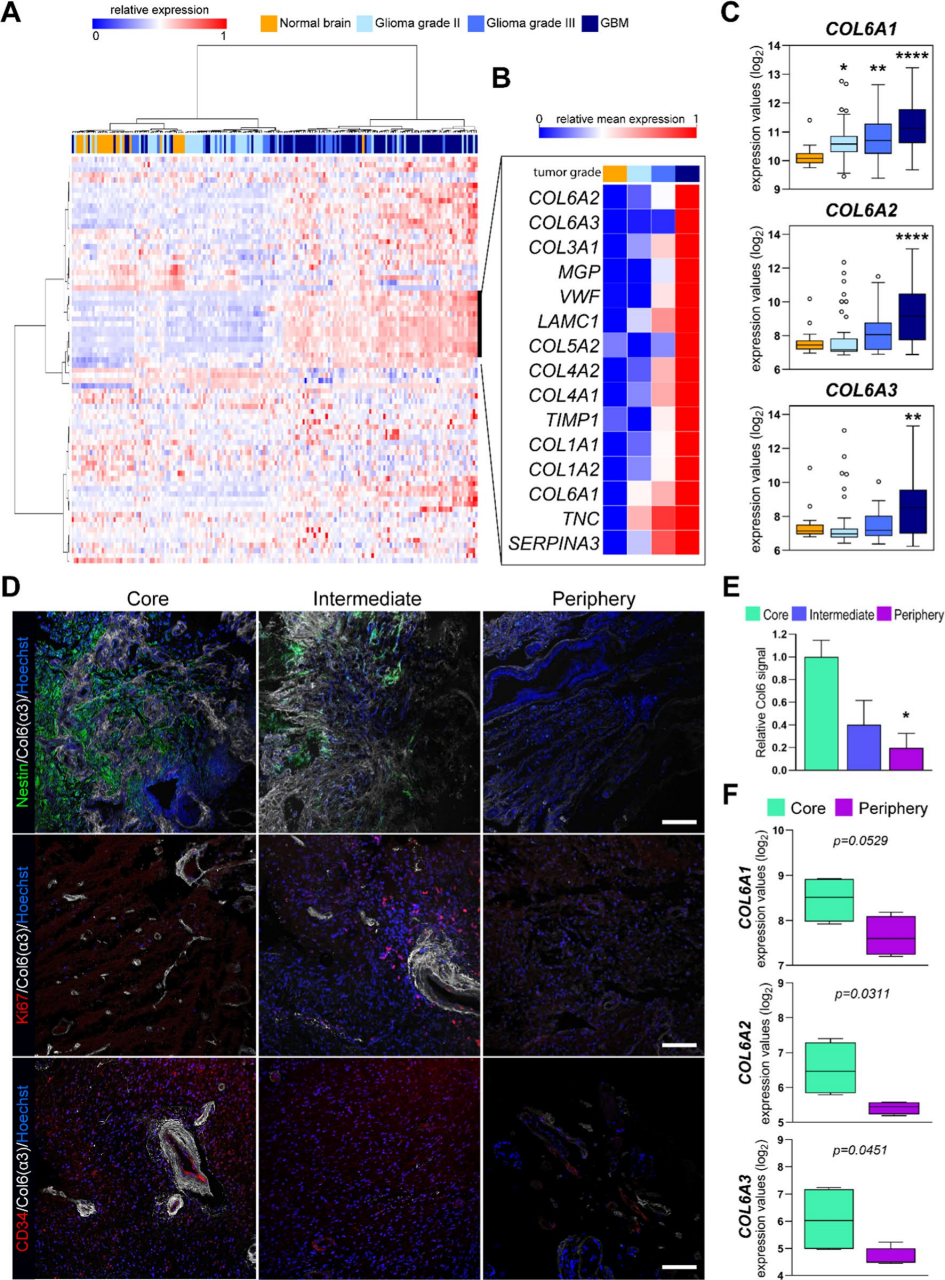
COL6 is overexpressed in GBM and displays a gradient pattern of intra-tumoral distribution. **A** Hierarchical analysis showing differential expression of the 79 selected ECM genes between high-grade gliomas and low-grade/normal brain samples from the GSE4290 dataset [45]. **B** Mean expression of a subset of 15 highly overexpressed genes in high-grade gliomas, extrapolated from the hierarchical analysis shown in (**A**). **C** Boxplots displaying the increasing levels of expression of *COL6A1*, *COL6A2*, and *COL6A3* genes depending on higher grade of glioma samples analyzed. Statistical analysis was performed by comparing glioma samples (grade II, *n* = 45; grade III, *n* = 31; GBM, *n* = 77) with normal brain (*n* = 23) through One-way ANOVA with Dunnett’s multiple comparisons test (*p < 0.05; **p < 0.01; ****p < 0.0001). **D** Representative confocal immunofluorescence images displaying COL6 protein in tumor biopsies isolated from the GBM core (left panels), tumor periphery (right panels), or an intermediate layer between them (central panels). α3(VI) labeling (white) is compared with Nestin (green; upper panels), Ki67 (red; middle panels), and CD34 (red; lower panels) stainings. Cell nuclei were counterstained with Hoechst (blue); scale bar, 100 μm. **E** Relative quantification of COL6 protein signal among the indicated GBM layers (obtained from 5 different patients). Statistical analysis by One-way ANOVA with Tukey’s multiple comparisons test (*p < 0.05). **F** Boxplots showing increased expression of *COL6A1*, *COL6A2*, and *COL6A3* genes in the GBM core *versus* more peripheral tissues (*n* = 4), based on our previously published GSE113512 dataset [52]. Statistical analysis by *t* test

Expression levels of ECM-related genes are able to predict clinical outcome of breast cancer patients, depending on the coordinated expression of diverse ECM proteins and enzymes [44]. Based on this premise, we analyzed the potential impact of ECM proteins on the aggressiveness of glioma tumors. To restrict our focus to ECM proteins, we refined the list of genes coding for ECM-related proteins reported in [44] to those genes coding for ECM proteins or even proteins (non-enzymes) with extracellular localization (*n* = 79, based on the available information; Suppl. Table S3). Hierarchical clustering analysis of glioma tissues from the GSE4290 dataset [45], performed on the basis of the expression of the selected 79 genes, identified two distinct subgroups of patients, one comprising the majority of normal and low-grade tumors, and the other one mostly composed by grade III–IV gliomas (Fig. 1A). Of note, a small subset of ECM genes (*n* = 15) displayed a consistent increased expression in high-grade glioma samples compared to lower grade tumors and normal brain samples (Fig. 1B and Suppl. Fig. S1A). Within collagen genes represented in this subset (9/15), we found that all the three main COL6 genes (*COL6A1, COL6A2, COL6A3*) [18] displayed an increased expression in malignant tumors, with a significant and consistent overexpression in GBM (Fig. 1C). Supporting these data, we confirmed a reproducible higher expression of all three COL6 transcripts with tumor grade (Suppl. Fig. S1B–D) in additional independent glioma/GBM tumor datasets (GSE7696, [46]; TCGA-GBM, [47]; CGGA [48]), pointing at a role for COL6 in sustaining the aggressiveness of high-grade gliomas, as already reported in several other tumors [17].

Finally, based on the most recent IDH mutation-driven classification of grade 4 tumors into GBM (IDH wildtype) and grade 4 astrocytomas (IDH mutated, endowed with a milder outcome) [49–51], we evaluated if any difference in term of expression of COL6 genes occurred in samples, according to the IDH status. Interestingly, COL6 genes displayed a consistent and significant decreased expression in IDH-mutated tumors across all grades (from the TCGA-GBM and CGGA datasets), in line with the suggested increase of COL6 expression with tumor malignancy, which was still observed in their IDH wildtype counterpart (Suppl. Fig. S2A, B). Along this line, we also verified the presence of any correlation between the expression of COL6 genes and the methylation status of the MGMT promoter, which nevertheless did not display any evident association with COL6 levels (Suppl. Fig. S2C).

### COL6 deposition follows a core-to-periphery gradient in the GBM mass

We previously demonstrated that GBM tumors are characterized by a highly necrotic and hypoxic core, enriched in chemotherapy-resistant GSCs, surrounded by less hypoxic hyper-proliferating cells (intermediate layer), and by peripheral highly vascularized tumor regions displaying a more differentiated and chemotherapy sensitive cell phenotype [5, 7, 8]. To further corroborate a potential correlation between COL6 and tumor aggressiveness, we checked for COL6 expression and deposition within the GBM tumor mass. Of note, immunofluorescence in GBM tumors, sampled according to our previously reported three-layer concentric model of GBM cell distribution [8, 36], showed a markedly higher COL6 protein deposition in the core of GBM mass, with a progressive decrease in the more peripheral regions (Fig. 1D, E). To confirm this result, we took advantage of the available transcriptional data that we previously obtained from both GBM core and periphery (GSE113512; [52]). Unsupervised analysis of the above selected 79 ECM genes demonstrated their differential expression between GBM core versus GBM periphery samples (Suppl. Fig. S3A), with *COL6A1*, *COL6A2,* and *COL6A3* transcripts being significantly overexpressed in the highly aggressive cells from the GBM core (Fig. 1F). Accordingly, the observed higher transcription of *COL6A1-3* genes in the GBM core was also validated through investigation of single-cell RNAseq data from the GSE84465 dataset [53], in which nonmalignant cells were filtered out (Suppl. Fig. S3B).

Intriguingly, other collagen proteins (i.e., Collagen IV and Collagen VII) resulted barely detectable or even not differentially accumulated across different GBM regions (Suppl. Fig. S3C), further supporting the potential involvement of COL6 in sustaining key hallmarks of GBM tumors.

### COL6 expression in GBM stem cells prevents their differentiation

**Fig. 2:**
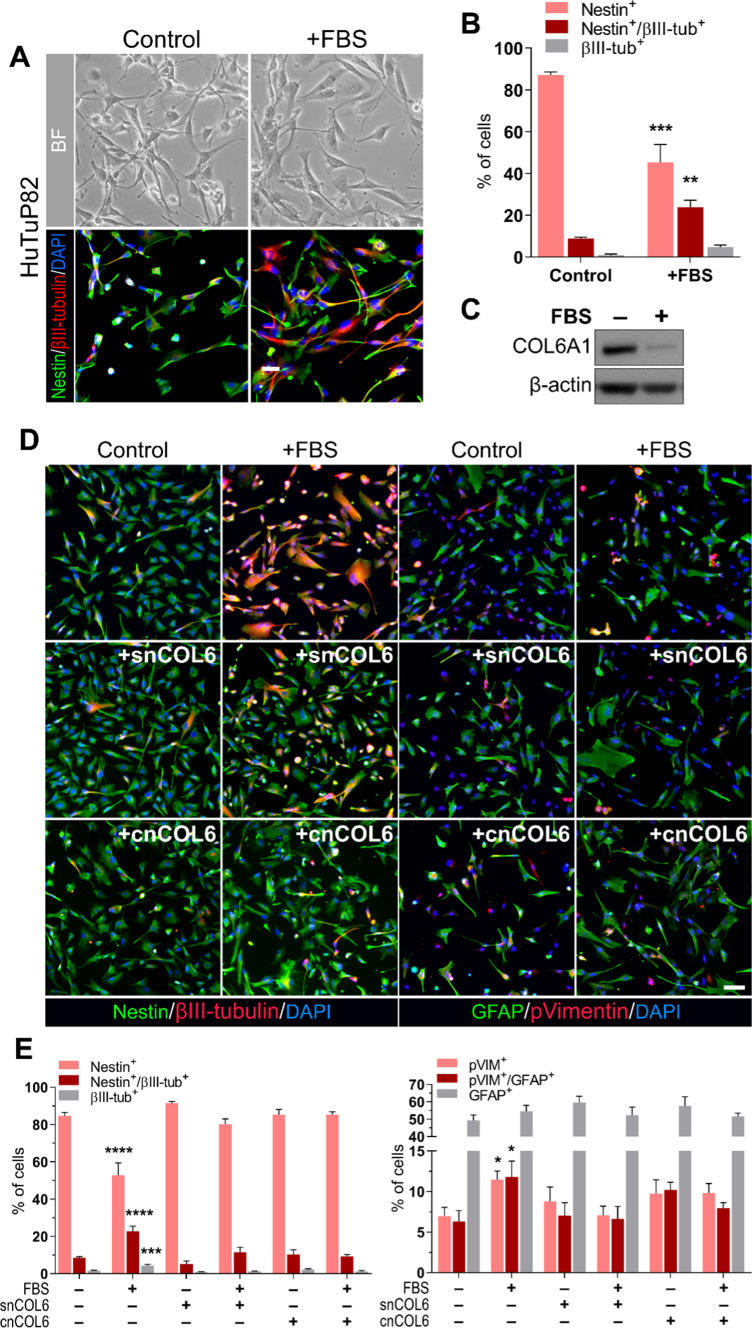
COL6 is overexpressed by GBM stem-like cells and prevents their differentiation. **A** Representative images displaying the morphological and phenotypic features of HuTuP82 GBM cells in undifferentiated conditions (control, left panels) and upon differentiation by 72 h exposure to 10% FBS-enriched medium (+ FBS, right panels). Top panels show bright field (BF) images, bottom panels display immunostaining for the neural stem cell marker Nestin (green) and the neuronal differentiation marker βIII-tubulin (red). Nuclei were counterstained with DAPI (blue). Scale bar, 20 μm. **B** Relative quantification of Nestin^+^/βIII-tubulin^–^, Nestin^+^/βIII-tubulin^+^ and Nestin^–^/βIII-tubulin^+^ GBM cells in samples as in (**A**). Statistical analysis by comparing FBS treated cells with controls through t test (*n* = 5; **p < 0.01; ***p < 0.001). **C** Representative western blot analysis for the α1(VI) COL6 chain (COL6A1) in HuTuP82 GBM cells in undifferentiated (– FBS) and differentiating (+ FBS) conditions. β-actin was used as a loading control. (**D**) Representative immunofluorescence images displaying the combined expression of Nestin (green) and βIII-tubulin (red) (left panels) or GFAP (green) and phospho-Vimentin (pVIM; red) (right panels) in HuTuP82 GBM cells under undifferentiated (control) and differentiating (72 h FBS) conditions, in the absence of any further treatment (top row) or in the presence of snCOL6 (1 μg/ml any other day; middle row) or of cnCOL6 (5 μg/cm^2^; bottom row). Nuclei were counterstained with DAPI (blue). Scale bar, 20 μm. **E** Relative quantification of Nestin^+^/βIII-tubulin^–^, Nestin^+^/βIII-tubulin^+^ and Nestin^–^/βIII-tubulin^+^ (left panel; *n* = 12) or of pVIM^+^/GFAP^–^, pVIM^+^/GFAP^+^, and pVIM^–^/GFAP^+^ (right panel; *n* = 8) in HuTuP53 and HuTuP82 GBM cells treated as in **D**. Statistical analysis was performed by comparing each experimental group with control cells (– FBS, – nCOL6) through One-way ANOVA with Dunnett’s multiple comparisons test (*p < 0.05; ***p < 0.001; ****p < 0.0001)

Cell populations residing in the inner regions of the GBM mass are enriched in stem-like cells and are exposed to noticeable hypoxia, as we already demonstrated [5]. Moreover, COL6 immunoreactivity in the GBM core is strictly associated with the neural stem cell marker Nestin (Fig. 1D, upper panels). Based on this evidence, we asked if COL6 overexpression could be dependent on microenvironmental low oxygen levels, or even expansion of the GSC compartment. In this context, the expression of COL6 genes remained unchanged when GBM cells were cultured under different oxygen tensions (Suppl. Fig. S4A), thus excluding a direct regulation by environmental oxygen levels. To explore a potential association of COL6 protein expression with GSCs, we pushed them towards differentiation by exposure to a 10% FBS-containing medium. Upon differentiation, beside the dramatic drop of Nestin expression and the acquisition of neuronal differentiation traits (Fig. 2A, B and Suppl. Fig. S4B, C), there was a dramatic reduction of COL6 protein levels (Fig. 2C and Suppl. Fig. S4D), suggesting a role of this ECM protein in the maintenance of a stem cell phenotype.

To verify this hypothesis, we exposed GBM cells to FBS, in the absence or presence of soluble native COL6 (snCOL6) and let them differentiate for 72 h. In agreement with the above findings, snCOL6 fully counteracted the FBS-induced differentiation by preventing the increase of βIII-tubulin^+^ cells and the expansion of a phospho-Vimentin (pVIM)^+^/GFAP^+^ glial progenitor differentiating cell population (Fig. 2D, E). Of note, the effects displayed by COL6 seemed to be elicited at the cell progenitor level, since we could not detect any modulation of the early stem cell marker Nanog (Suppl. Fig. S4E). Highly comparable results were obtained when the same experiments were performed with GBM cells plated onto nCOL6-coated (cnCOL6) plates (Fig. 2D, E). From a functional point of view, although native COL6 did not modulate the already highly immature phenotype of primary GBM cultures by itself, its administration was sufficient to significantly enhance their self-renewing capacity in both basal and pro-differentiating conditions (Suppl. Fig. S4F).

To further understand how COL6 exerts the observed effect on GBM cell differentiation, we compared data obtained using the native protein (nCOL6) with a pepsin-resistant fragment of COL6 (pepCOL6), which solely retains the triple helical domain. Interestingly, exogenous addition of pepCOL6 failed to fully counteract FBS-induced differentiation (Suppl. Fig. S5A, B), therefore suggesting that the presence of the N- and C-terminal globular regions of COL6 is required to achieve a completely functional pro-stemness/anti-differentiation activity.

Altogether, these results indicate that COL6 plays a major role in modulating the balance between cell differentiation and stem-like cell maintenance, by sustaining the preservation of both functional and phenotypic stemness in GBM.

### COL6 silencing significantly affects GBM cell differentiation

**Fig. 3:**
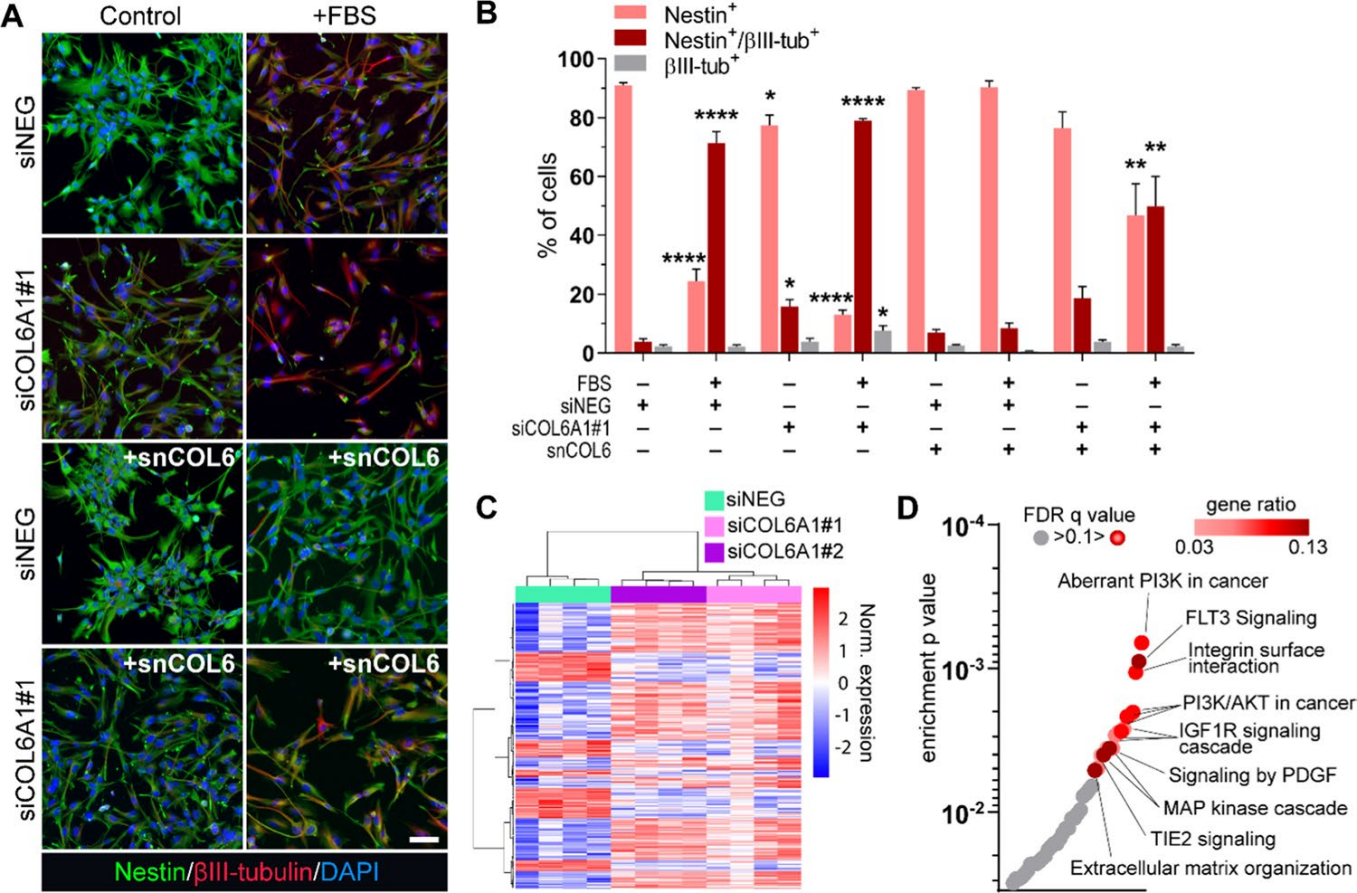
COL6 silencing affects GBM cell differentiation and gene transcription. **A** Representative co-immunofluorescence images for Nestin (green) and βIII-tubulin (red) in siNEG or siCOL6A1#1 transfected GBM cells under control and differentiating (72 h FBS) conditions, in the absence of any further treatment or in the presence of snCOL6 (1 μg/ml any other day). Nuclei were counterstained with DAPI (blue). Scale bar, 20 μm. **B** Relative quantification of Nestin^+^/βIII-tubulin^–^, Nestin^+^/βIII-tubulin^+^ and Nestin^–^/βIII-tubulin^+^ HuTuP82 GBM cells as in **A**. Statistical analysis was performed by comparing each experimental group (*n* = 3) with control siNEG cells (– FBS, – snCOL6) through One-way ANOVA with Dunnett’s multiple comparisons test (*p < 0.05; **p < 0.01; ****p < 0.0001). **C** Heatmap summarizing differentially expressed genes between siNEG, siCOL6A1#1 and siCOL6A1#2 transfected HuTuP82 GBM cells. **D** Dot plot summarizing the significant (shades of red) negatively enriched pathways in COL6-silenced HuTuP82 GBM cells by over-representation analysis of the C2cp gene sets

Since COL6 administration did not further enhance the stem cell phenotypic traits, already highly enriched in GBM cells in basal culturing conditions (Fig. 2), we wondered if COL6 deficiency could negatively affect GBM stemness and even potentiate any induced differentiation. To this end, we silenced COL6 expression using a siRNA (siCOL6A1#1) targeting *COL6A1* (Suppl. Fig. S5C), whose ablation is known to prevent the assembly of the functional COL6 protein [18, 39], evaluating its impact in GBM behavior in both resting and pro-differentiating conditions. We found that COL6 silencing partially, although significantly, reduced stemness and increased neuronal differentiation of GBM cells, reducing and increasing the expression of Nestin and βIII-tubulin, respectively, relative to control cells (Fig. 3A, B). A similar induction of a pVIM^+^/GFAP^+^ progenitor cell population was also observed (Suppl. Fig. S5D, E). Intriguingly, administration of snCOL6 largely counteracted COL6 silencing effects in pro-differentiating, FBS-dependent conditions (Fig. 3A, B).

These data support the concept that endogenously produced COL6 is required to maintain GBM cell stemness and that the achievement of a threshold in COL6 expression is crucial for counteracting environmentally induced differentiation.

### COL6 suppression significantly affects a cancer-related aberrant gene transcription

To explore the intracellular processes affected by COL6 in GBM, we analyzed the transcriptional changes of GBM cells after COL6 silencing (Suppl. Fig. S6A). To filter our data for potential off-target effects, we considered only transcripts that were commonly perturbed by both COL6 siRNAs, identifying 169 up-regulated and 84 down-modulated genes upon COL6 abrogation (Fig. 3C, Suppl. Fig. S6B and Suppl. Table S4). Of note, GBM cells did not display any potential transcriptional compensatory mechanism engaged in response to COL6 silencing, since we could not detect any increased expression of other collagen or ECM genes at the considered timepoints (Suppl. Table S4).

Enrichment analysis disclosed downregulated genes as significantly involved in several cancer associated intracellular pathways including PI3K/AKT, IGF1, FLT3, PDGF, and MAPK, together with microenvironmental processes such as ECM and TIE2 signaling (Fig. 3D). On the other hand, analysis of upregulated genes did not produce relevant significant transcriptional enrichments, with the exception for the Ca^2+^ and mTOR signaling pathways (Suppl. Fig. S6C), which were already reported to be positively affected by COL6 ablation in other non-cancerous contexts [54, 55].

These results further support the involvement of COL6 in sustaining GBM cell aggressiveness and are in agreement with functional data demonstrating its role in modulating stem-like cell plasticity and phenotype.

### A COL6-dependent transcriptional module is involved in chemotherapy response and patient outcome

**Fig. 4:**
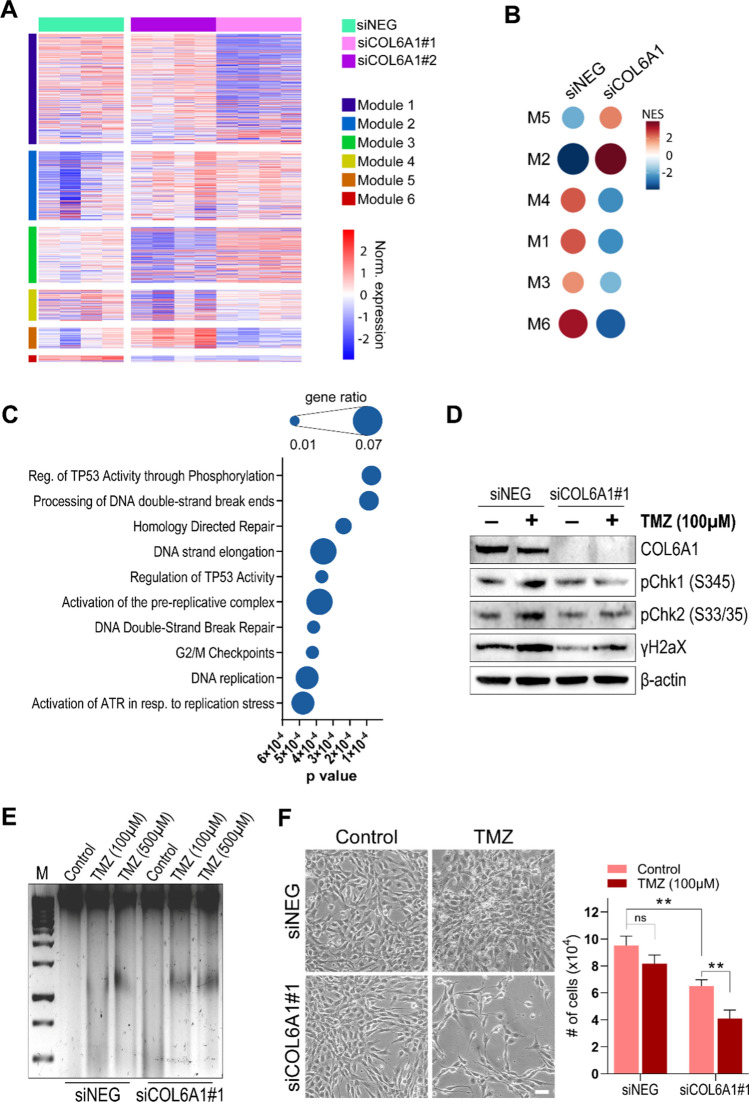
COL6 correlated genes sustain chemotherapy resistance in GBM cells. **A** Heatmap displaying the identification of six different transcriptional modules, each comprising genes with coordinated expression across HuTuP82 GBM cells transfected with siNEG, siCOL6A1#1 or siCOL6A1#2. **B** Dotplot summarizing normalized enrichment of the six transcriptional modules (M1–M6) in siNEG and siCOL6A1 subgroups (both #1 and #2) of samples. NES: Normalized Enrichment Score. **C** Dotplot showing pathway enrichments of the 35 genes belonging to the M6 transcriptional module. **D** Representative western blot analysis for α1(VI) COL6 chain (COL6A1), phospho-Chk1, phospho-Chk2, and γH2aX in siNeg or siCOL6A1#1 transfected HuTuP82 GBM cells in the absence (–) or presence (+) of 100 μM TMZ stimulation for 24 h. β-actin was used as a loading control. **E** Representative electrophoresis displaying DNA damage products derived from HuTuP82 GBM cells transfected with siNeg or siCOL6A1#1 under control conditions or upon 24 h TMZ treatment at 100 μM or 500 μM. Size marker (M) is shown on the left. **F** Representative brightfield images of HuTuP82 GBM cells transfected with siNeg or siCOL6A1#1 under control conditions or upon 72 h exposure to 100 μM TMZ. The histogram on the right shows the trypan blue-based quantification of growth kinetics in the different conditions. Scale bar, 20 μm (**p < 0.01; ns, not significant; Student’s *t* test)

Based on the above described COL6-dependent modulation of several relevant pathways in GBM cells, we investigated if a COL6 transcriptional signature could be inferred and then characterized for its potential role in the control of any additional cancer hallmark. To this end, we explored our transcriptional data to identify distinctive subsets of genes displaying a significant and consistent correlated expression within the siNEG or the siCOL6A1(#1/#2) samples. Then, by subdividing identified genes into co-expression clusters, we identified 6 different transcriptional modules (M1-6) with a coordinated and significant differential expression among the siNEG and siCOL6A1 samples (Fig. 4A). *COL6A1* resulted consistently co-expressed among the experimental groups (siNEG vs siCOL6A1) with other 34 genes, together belonging to the M6 transcriptional module. Intriguingly, this gene cluster resulted as the most negatively enriched in COL6-silenced samples by gene set enrichment analysis (GSEA) (Fig. 4B). These data confirmed the presence of a specific subset of genes whose expression could be immediately dependent (directly or indirectly) on COL6 expression/deposition. Moreover, over-representation pathway analysis of the M6 cluster disclosed that these genes have a major role in the repair of DNA damage, surveillance of DNA integrity at the level of cell cycle checkpoints, and control of DNA replication processes under stressful conditions (Fig. 4C).

In order to functionally confirm the above results, we silenced *COL6A1* in GBM cultures and then exposed them to TMZ, the gold standard chemotherapeutic administered to glioma patients [2]. COL6 suppression was sufficient to prevent the activation of the ATR/ATM axis downstream effectors Chk1, Chk2, and γH2aX (Fig. 4D), which are generally activated by cells in response to DNA damaging stimuli in order to promote DNA repair [56]. On the other hand, since HuTuP82 cells bear a R248W mutation affecting DNA damage-triggered p53 response [57], we could not detect any change in p53 phosphorylation upon TMZ treatment or COL6 suppression (data not shown). Functionally, TMZ-induced DNA damage was enhanced by COL6 silencing (Fig. 4E), which also significantly strengthened the very limited antiproliferative effects exerted by TMZ (Fig. 4F).

**Fig. 5:**
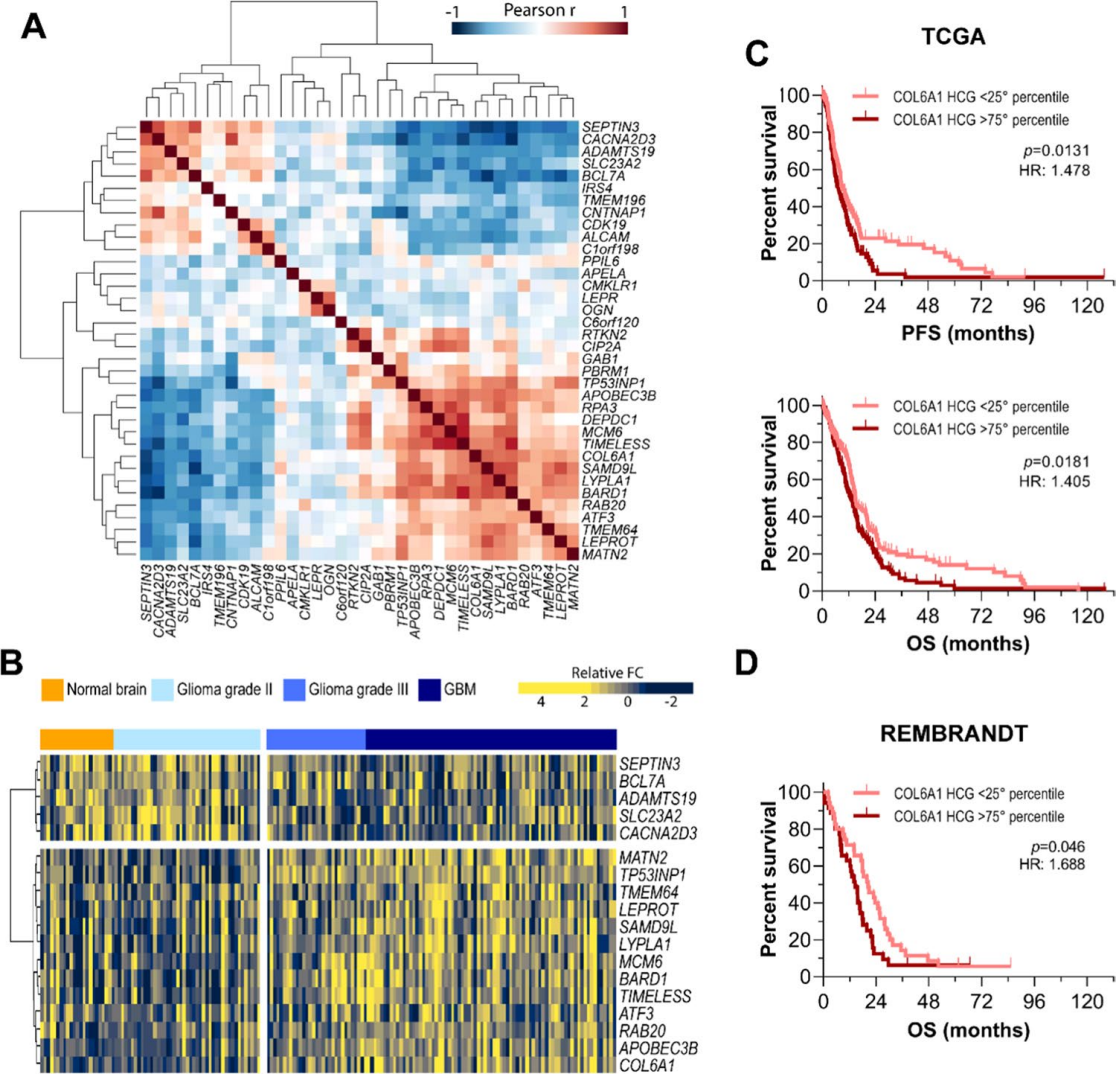
A COL6-dependent gene signature correlates with a worse GBM patient outcome. **A** Heatmap displaying Pearson *r* correlation values of the 35 genes belonging to the M6 transcriptional module in glioma samples from the GSE4290 dataset. The expression of Highly Correlated Genes (HCG) across glioma samples (both positively and negatively correlated, *r* > 0.4 and *r* < − 0.4, respectively) is shown in (**B**). *FC* Fold Change. Progression-free survival (PFS) and overall survival (OS) curves of GBM patients from the TCGA (*n* = 519, **C**) and the Rembrandt (GSE68848) (*n* = 181, **D**) datasets, sub-grouped on the basis of the cumulative mean-centered expression of highly correlated genes (*r* >  0.4) selected from **B**. Statistical analysis by Log-rank (Mantel-Cox) test. *HR* hazard ratio

Based on these results, we verified if such *COL6A1*-orchestrated transcriptional signature could have a clinical impact in terms of tumor malignancy and patient survival. We first evaluated whether genes belonging to the M6 module displayed a correlated expression in patient samples from the GSE4290 dataset. This analysis showed that more than 70% of M6 genes were highly correlated with each other, either negatively or positively, in glioma tumors (Fig. 5A), confirming that at least a subset of the previously identified *COL6A1*-correlated genes displays a similar behavior also in tumor samples from patients. Of note, genes displaying a strong negative correlation (*r* < − 0.4; *n* = 5) with *COL6A1* were particularly expressed in both normal brain samples and low-grade gliomas relative to higher grade tumors. Conversely, the expression of *COL6A1* and its highly positively correlated genes (*r* ≥ 0.4; *n* = 12) increased progressively with tumor grade/malignancy (Fig. 5B), suggesting once again that COL6, possibly through a coordinated action with a series of strictly correlated genes, may have a critical role in sustaining GBM severity. Moreover, analyzing the expression of *COL6A1* and its correlated genes in GBM samples from the TCGA [58] or the Rembrandt (GSE68848; [59]) datasets, we found that they negatively impact on GBM patient prognosis, with patients expressing high levels of these genes (> 75° percentile) being characterized by a significant worse progression and overall survival (Fig. 5C, D), in line with a predictable higher malignancy when COL6, and the co-regulated genes are upregulated in GSC cells. These data suggest that COL6, besides acting as a potent modulator of GSC differentiation capabilities, may also affect the expression of a peculiar subset of genes involved in the maintenance of DNA integrity, eventually impacting not only on GBM cell response to chemotherapy in vitro, but also on GBM patient outcome, with relevant implications for the future investigations in the field**.**

## Discussion

In this work, we demonstrate that COL6 represents a microenvironmental hallmark of aggressiveness in GBM tumors by exerting a diverse, nevertheless highly integrated, set of pro-cancerous effects including, but possibly not limited to (i) supporting the maintenance of an immature GSC-like phenotype of cancer cells, by preventing their environmentally induced differentiation; (ii) enhancing the activation of a malignant transcriptional program; and (iii) promoting, in a coordinated way with a set of highly correlated genes, an efficient cellular response (in terms of DNA repair) to replicative stress and DNA-damaging agents. These findings are in line with the previously suggested higher expression of COL6 in high grade gliomas [26, 34, 60, 61] and its known involvement in the biology of several types of cancer [62].

Besides the already described role of COL6 in the satellite cell niche of skeletal muscles, where it sustains self-renewal and tissue regeneration [24], the proper expression and ECM deposition of COL6 in the nervous system has been recently linked to the ability of several brain compartments, including blood vessels and meninges, but also neurons and glial cells, to correctly develop and differentiate into mature functional entities [63, 64]. Our data demonstrate that COL6 plays a pivotal role also in the brain tumor context, supporting the generation of a favorable microenvironment for GSC maintenance. Intriguingly, we found that, in COL6-silenced cells, exogenously COL6 reintegration is no longer sufficient to counteract GSC differentiation. Although we cannot exclude that a progressive increase of microenvironmental COL6 levels may potentially restore such an effect, our results point at a cell-autonomous role for COL6 expression in cancer stem cells. Indeed, the specific role of COL6 endogenously produced by cancer stem cells was poorly investigated thus far in literature studies, whereas much more efforts were spent in exploring the role and mechanisms by which COL6 secreted by tumor supporting cells—mainly fibroblasts and adipocytes—acts as a pivotal environmental cue affecting tumor progression and aggressiveness in different contexts, with strong impact in mammary tumor cells [31, 65, 66], but also in pancreatic ductal adenocarcinoma [25, 67], and ovarian cancer [68]. Since COL6 was shown to serve as a chemoattractant for macrophage recruitment and even polarization toward an M2 suppressive phenotype during tissue regeneration [29], its potential involvement should be also considered in generating a favorable milieu further contributing to an eased tumor cell recovery, progression, and expansion after treatments, eventually assisting relapse [69]. Nonetheless, recent works highlighted how the suppression of endogenously produced COL6 strongly affects migration and invasion of pancreatic [70] and breast cancer cells, here impairing mammosphere formation capacity, representative of a reduction in breast tumor stem-like cell phenotype [71]. In this context, further studies will be needed to clarify whether a progressive increase in COL6 concentrations would possibly go beyond a specific threshold, thus counteracting external stimuli-induced differentiation in COL6-silenced cells.

As an extracellular protein able to bind several cell surface receptors [18], it is conceivable that COL6 exerts its effects in counteracting GSC differentiation by engaging with membrane receptors, thus triggering intracellular signaling cascades. While the nature of such interaction was not investigated in this work, N- and C-terminal globular domains are expected to mediate a major impact, since pepCOL6 failed to fully counteract FBS-induced differentiation. Interestingly, COL6 interaction with integrins α1β1 and α2β1 was reported to occur via its triple helical domain [72], and the same was reported to occur with ANTXR2/CMG2 [73], but not with ANTXR1/TEM8, as the latter instead was found to contact the C5 terminal domain of COL6A3 [74]. Conversely, another COL6 receptor, the chondroitin sulfate proteoglycan CSPG4/NG2, was described to bind both the triple helical region and the terminal globular regions of COL6 [62].

In our study, silencing of COL6 in GBM cells induced the downregulation of genes associated with cancer-relevant pathways, including PI3K/AKT, IGF1, FLT3, PDGF, and MAPK pathways. Of note, a previous work demonstrated that COL6 is able to activate AKT signaling through the NG2/CPSG2 receptor expressed on the plasma membrane of breast cancer cells, in turn promoting tumor growth [65]. Interestingly, AKT phosphorylation was shown to be similarly affected upon *COL6A3* siRNA-mediated silencing in osteosarcoma cells [75], also displaying a marked deregulation upon *COL6A1* and *COL6A2* modulation in bladder cancer cells [76]. Beside COL6 ability to modulate the PI3K/AKT pathway in cancer, in vivo and in vitro studies demonstrated an impact of COL6 on such axis in other districts, with also deregulation of MAPK/ERK kinases and upregulation of mTOR in the COL6 knockout context [21, 77]. Within our present study, together with upregulation of genes associated with mTOR, Ca^2+^ signaling also emerged as positively enriched by GSEA upon *COL6A1* siRNA, in line with recent evidence linking COL6 deficiency with altered Ca^2+^ permeability, due to upregulation of STIM1 and ORAI1 [54].

Our data strongly support a role for COL6 in sustaining the processes of intact cell replication and of DNA remodeling and repair in GBM cells. Indeed, our analyses showed that a characteristic gene signature, transcriptionally highly correlated to COL6 expression in both COL6-silenced cells and GBM patients, is involved in enhancing cell ability to repair chemotherapy-induced DNA damage, thus providing a tolerant response to replicative stress and enhancing an aberrant activation of multiple pro-survival signaling pathways. Moreover, we found that the observed increased expression of COL6 in high-grade gliomas, as also reported in other studies [78, 79], significantly impacts on GBM patients’ prognosis, possibly acting in a coordinated manner together with DNA replication and repair genes. In this context, we should also consider the plausible contribution of COL6-dependent phenotypic control in sustaining such a pro-malignant GBM cell behavior.

Recent studies clearly highlighted that ECM proteins, including different collagens, stimulate a distinctive integrin-dependent intracellular signaling activation in several cancer contexts—GBM included—eventually sustaining DNA repair and cell survival in response to both radio- and chemotherapy [80–82]. Therefore, it is not implausible that a similar COL6-dependent activation of specific DNA repair machineries may be sensed by GBM cells through specific integrins. In line with this, the recent literature work showed that chemotherapy treatment is able to induce and modulate matrisome expression, and in particular that of COL6 genes, in primary and metastatic ovarian carcinoma, enhancing tumor resistance to treatments by promoting integrin binding to a COL6 substrate [83]. However, further studies will be needed in order to decipher the molecular players participating in the COL6 interactive network able to transduce these signals, and to characterize their individual impact on cell signaling activation and gene transcription. Although the therapeutic targeting of major ECM components—such as COL6—would be quite challenging, due to the expected widespread side-effects in multiple organs of the body, approaches based on the targeted inhibition of unique extracellular or intracellular ECM-dependent interactions warrant further consideration for the setup of adjuvant sensitizing treatments [80] able to provide a more efficient cancer cell eradication and even prevent tumor relapse. Along this line, antibody-based targeted approaches were preliminarily tested in simplified in vivo or in vitro models, providing the proof-of-concept that (i) extracellular COL6 can represent an “accessible” target for intravenously administered antibody-based therapy [35] and (ii) specific inhibition of COL6-mediated signal transduction can increase sensitivity to chemotherapy [33].

Previous studies demonstrated that *COL6A1* expression is correlated to a metastatic behavior in pancreatic cancer [70] and may also contribute to the process of brain metastasis in breast tumors [84]. Moreover, increased levels of COL6 or its cleavage product ETP [31] were shown to be positively correlated to an increased resistance to cisplatin-based therapies in non-small cell lung cancer cell lines and patients, and in breast cancer mouse models, respectively [33, 85]. Nevertheless, little is known about the abundance of COL6 in relapsed tumors. Recent work suggested that COL6 is increased in patients’ metastases after chemotherapy and provides enhanced adherence and resistance to ovarian cells from relapses upon in vitro cisplatin treatment [83]. Based on our results, we can hypothesize that a COL6-enriched cancer niche may provide a protective environment for therapy-resistant GBM stem-like cells, pointing at a tumor-supportive effect for COL6 accumulation in recurrent tumors. Despite a preliminary exploration of available mRNA expression databases portrays only a slight, although significant, increased transcription of COL6 genes in GBM relapses (Suppl. Fig. S7), a thorough and more detailed characterization of COL6 protein levels in recurrent GBM may provide further knowledge on the molecular mechanisms associated with their fast onset and recognized therapeutic insensitivity, with relevant implications on the future definition of the role of COL6 in TMZ (or in more general terms chemotherapy) sensitivity.

### Supplementary Information

Below is the link to the electronic supplementary material.

Supplementary file1 (PDF 1398 KB)

Supplementary file2 (XLSX 17 KB)

## Data Availability

Expression data have been deposited into the Gene Expression Omnibus (GEO) database under Series Accession Number GSE226700 and are accessible without restrictions. Additional results were generated by re-analysis of publicly available data provided by the GSE4290 [45], GSE7696 [46], GSE84465 [53], TCGA-GBM [47], CGGA [48], TCGA (U133A) [58], GSE68848 [59], and GSE113512 [52] datasets.
