# Supplementary material for: Collagen VI sustains cell stemness and chemotherapy resistance in glioblastoma
Source: Cell Mol Life Sci. 2023 Jul 28;80(8):233. doi: 10.1007/s00018-023-04887-5 (PMC10382393; doi:10.1007/s00018-023-04887-5)
Supplement: Supplementary file 1 — Supplementary file1 (PDF 1398 KB) [file 18_2023_4887_MOESM1_ESM.pdf]

Collagen VI sustains cell stemness and chemotherapy resistance in glioblastoma.

Cellular and Molecular Life Sciences

Matilde Cescon, Elena Rampazzo, Silvia Bresolin, Francesco Da Ros, Lorenzo Manfreda, Alice Cani, Alessandro Della Puppa, Paola Braghetta, Paolo Bonaldo, and Luca Persano.

Correspondence to:

Luca Persano, PhD

email: luca.persano@unipd.it

Ph: +390498215487

Department of Women and Children's Health, University of Padova

via Giustiniani 3, 35127 Padova, Italy.

SUPPLEMENTARY INFORMATION

Supplementary Table S1. GBM specimens and primary cultures used within the study. All samples are classified as IDH wildtype.

Patient ID	Tissue	Primary cells	Diagnosis	WHO grade	Gender	Age (yr)
**HuTuP53**	N	Y	GBM	IV	M	69
**HuTuP56**	Y	N	GBM	IV	M	46
**HuTuP80**	Y	N	GBM	IV	M	55
**HuTuP82**	Y	Y	GBM	IV	M	49
**HuTuP101**	Y	N	GBM	IV	M	64
**HuTuP108**	Y	N	GBM	IV	M	62
**HuTuP148**	Y	N	GBM	IV	M	81

M, male; N, no; Y, yes; yr, years.

Supplementary Table S2. List of primer sequences used within the study.

Gene	***Sequence (5'-3')***	***Amplicon (bp)***
COL6A1*, forward**<br>COL6A1*, reverse**	*CCCGTGGACCTGTTCTTTGT*<br>*CACAGCGGTAGTACCTGTCC*	139
COL6A2*, forward**<br>COL6A2*, reverse**	CTCGGGACCAGGACTTCAG<br>GGCAGTTGTTGTTTCTCTCGG	150
COL6A3*, forward**<br>COL6A3*, reverse**	GCCCATGGTTAAGATGTCCCG<br>GAGGTGACGGTGAGGTCATAAAA	120
CA9*, forward**<br>CA9*, reverse**	CAGTTGCTGTCTCGCTTGGA<br>TCAGAGGGCAGGAGTGCA	89
GUSB*, forward**<br>GUSB*, reverse**	GAAAATACGTGGTTGGAGAGCTCATT<br>CCGAGTGAAGATCCCCTTTTTA	101

bp, base pairs.

Supplementary Table S3. List of 79 ECM genes selected from the 278 ECM-related genes used in the study from Bergamaschi *et al.* [1].

COL1A1	*COL9A1*	*FBLN5*	*LAMB2*	*SERPINA7*	*TNR*
COL1A2	*COL9A2*	*FBN1*	*LAMB3*	*SERPINA10*	*TNXB*
COL2A1	*COL9A3*	*FBN2*	*LAMC1*	*SLPI*	*VTN*
COL3A1	*COL12A1*	*FBN3*	*LAMC2*	*SMOC2*	*VWF*
COL4A1	*COL14A1*	*FCN1*	*LEPRE1*	*SPARC*	
COL4A2	*COL15A1*	*HABP2*	*MATN1*	*SPARCL1*	
COL4A5	*COL16A1*	*HAPLN2*	*MGP*	*SPINK4*	
COL4A6	*COL18A1*	*HSPG2*	*PI3*	*SPINK5*	
COL5A2	*COL21A1*	*IMPG1*	*PI15*	*SPINT1*	
COL5A3	*COMP*	*ITGBL1*	*PI16*	*SPOCK2*	
COL6A1	*ECM1*	*LAMA2*	*PRG2*	*SPOCK3*	
COL6A2	*EMILIN1*	*LAMA3*	*SDF2*	*SPON1*	
COL6A3	*EMILIN2*	*LAMA4*	*SERPINA3*	*SPON2*	
COL7A1	*FBLN1*	*LAMA5*	*SERPINA5*	*TIMP1*	
COL8A2	*FBLN2*	*LAMB1*	*SERPINA6*	*TNC*	

Supplementary Figure S1

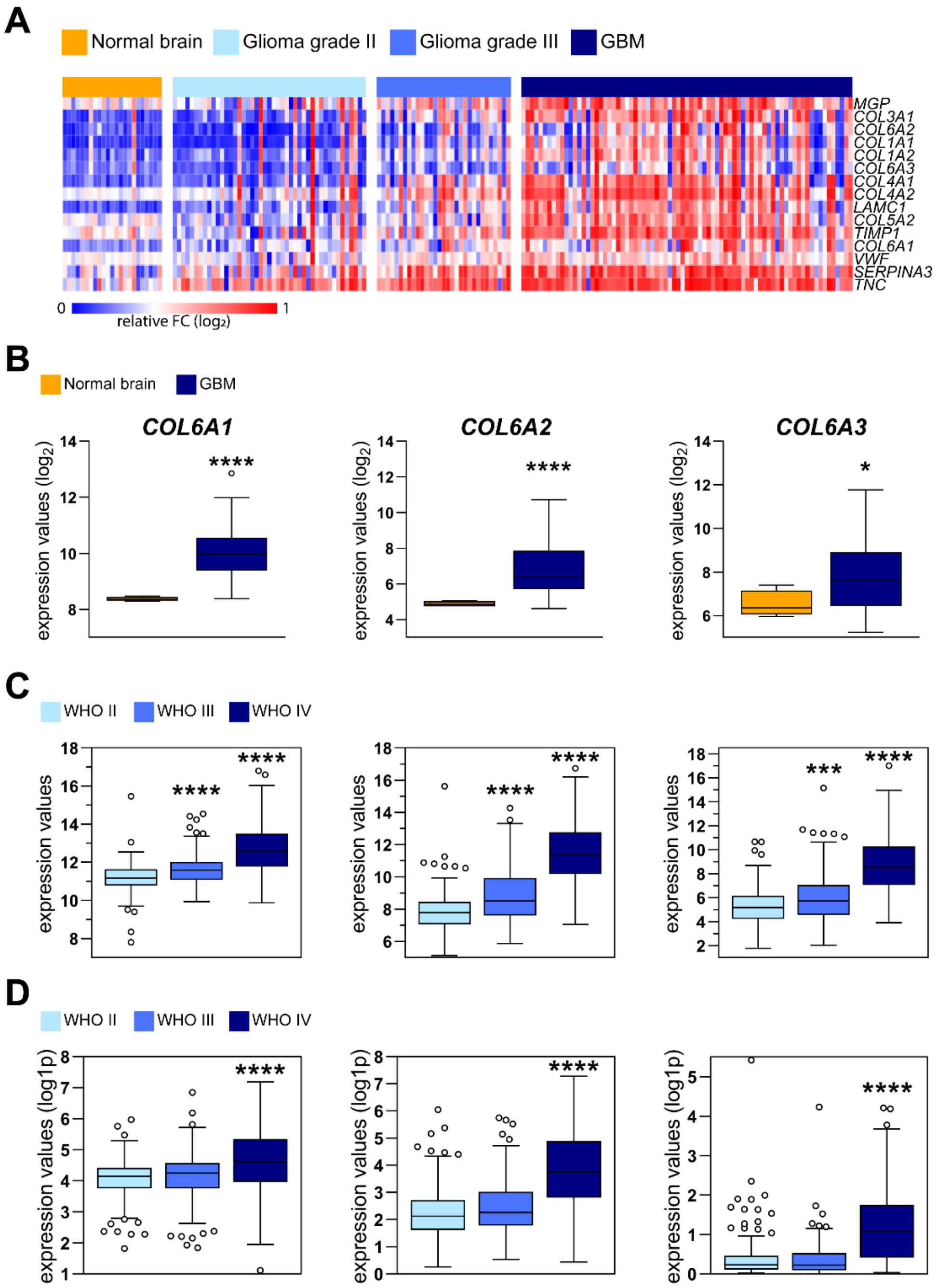

Supplementary Figure S1. COL6 genes are overexpressed in GBM and aggressive GBM subpopulations. (**A**) Heatmap showing expression of the selected ECM genes (as in Figure 1A, B) in individual patient samples from the GSE4290 dataset [2]. (**B-D**) Boxplots reporting the expression of *COL6A1* (left column), *COL6A2* (middle column) and *COL6A3* (right column) genes in: GBM (*n*=70) and normal brain samples (*n*=4) from the GSE7696 GBM patient dataset [3] (**B**); primary WHO II (*n*=216) and WHO III (*n*=241) gliomas, and WHO IV GBM (*n*=152) samples from the TCGA-GBM dataset [4] (**C**); primary WHO II (*n*=138) and WHO III (*n*=144) gliomas, and WHO IV GBM (*n*=140) samples from the CGGA dataset [5] (**D**). Statistical analysis by t test (**B**) and one-way ANOVA (**C**, **D**) with Tukey's multiple comparisons test (*, *p* <0.05; ***, *p* <0.001; ****, *p* <0.0001).

Supplementary Figure S2

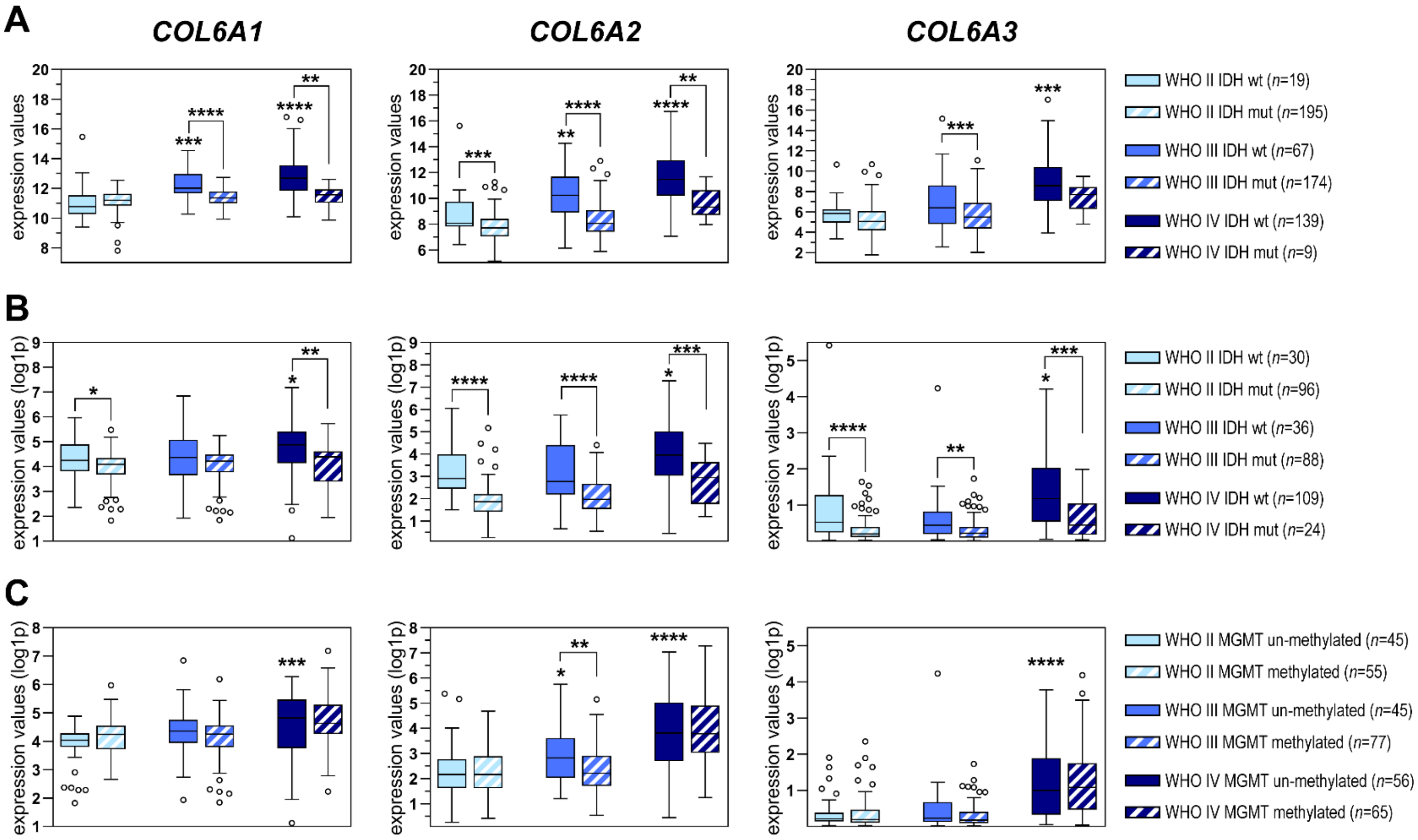

Supplementary Figure S2. Expression of COL6 genes across glioma samples with differential IDH mutational status and MGMT promoter methylation. (**A**, **B**) Boxplots reporting the expression of *COL6A1* (left column), *COL6A2* (middle column) and *COL6A3* (right column) genes in IDH wildtype or mutated primary glioma tumors (WHO II-IV) from the TCGA-GBM [4] (**A**) and the CGGA [5] datasets (**B**). (**C**) Boxplots reporting the expression of *COL6A1* (left column), *COL6A2* (middle column) and *COL6A3* (right column) genes in MGMT promoter un-methylated or methylated glioma tumors (WHO II-IV) from the CGGA dataset. Asterisks over the brackets indicate statistical significance between indicated samples (t test); asterisks over bars indicate statistical significance between WHO III or IV vs WHO II glioma samples belonging to the IDH wildtype or MGMT promoter un-methylated subgroups (one-way ANOVA with Tukey's multiple comparisons test). *, *p* <0.05; **, p <0.01; ***, *p* <0.001; ****, *p* <0.0001. wt, wildtype; mut, mutated.

Supplementary Figure S3

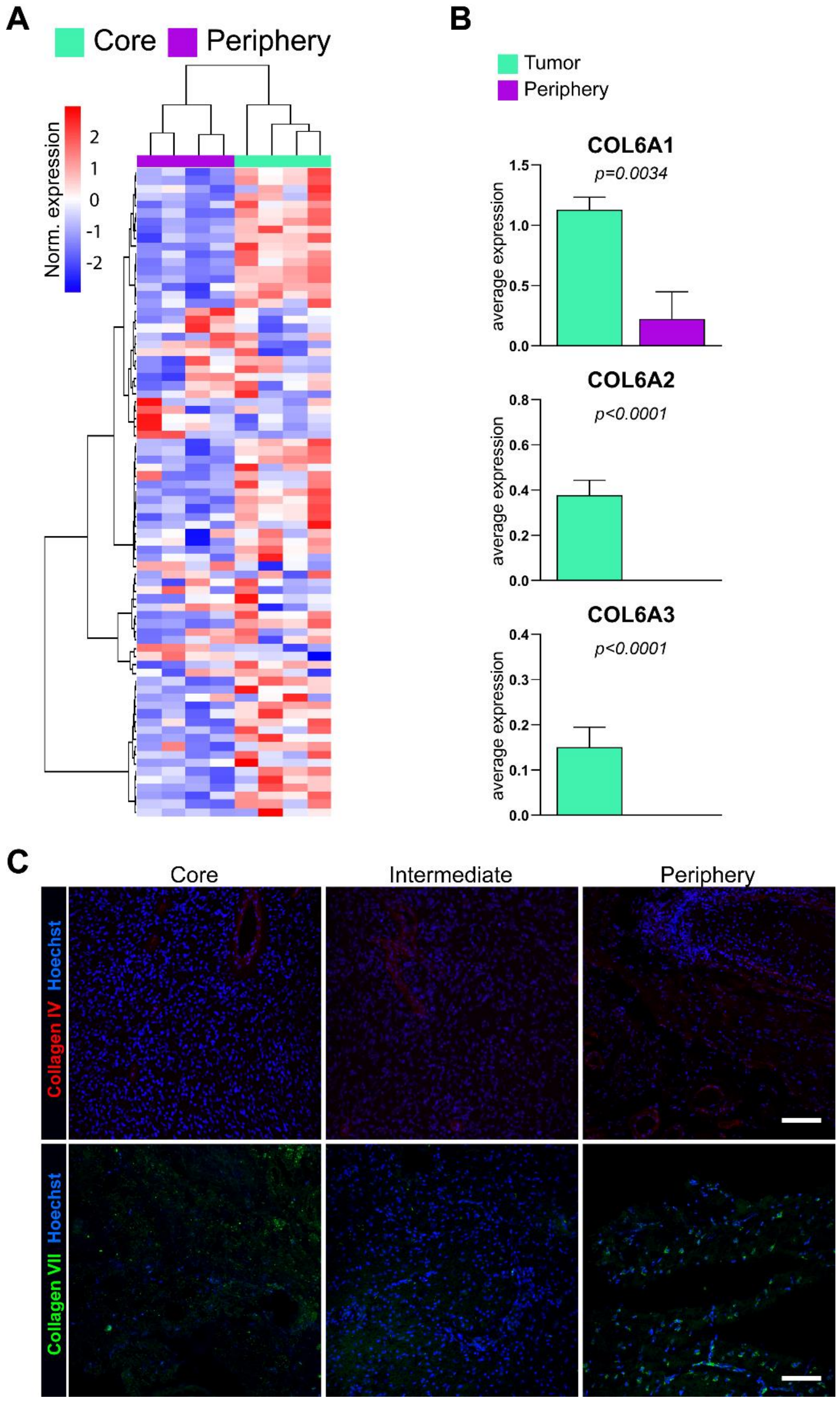

Supplementary Figure S3. Intratumoral distribution of ECM components. (**A**) Hierarchical analysis showing a differential expression of the selected 79 ECM genes (as in Figure 1A, B) between GBM core and periphery (*n*=4) from the GSE113512 dataset [6]. (**B**) Barplots showing increased expression of COL6 genes in malignant GBM cells retrieved from the tumor mass *vs* more peripheral tissues, based on single cell RNAseq data from the GSE84465 dataset [7]. Statistical analysis by t test. (**C**) Representative confocal immunofluorescence images of Collagen IV (red, upper panels) and Collagen VII (green, lower panels) in tumor biopsies isolated from the GBM core, tumor periphery, or an intermediate layer between them. Cell nuclei were counterstained with Hoechst (blue). Original magnification: 10X; scale bar, 100 µm.

Supplementary Figure S4

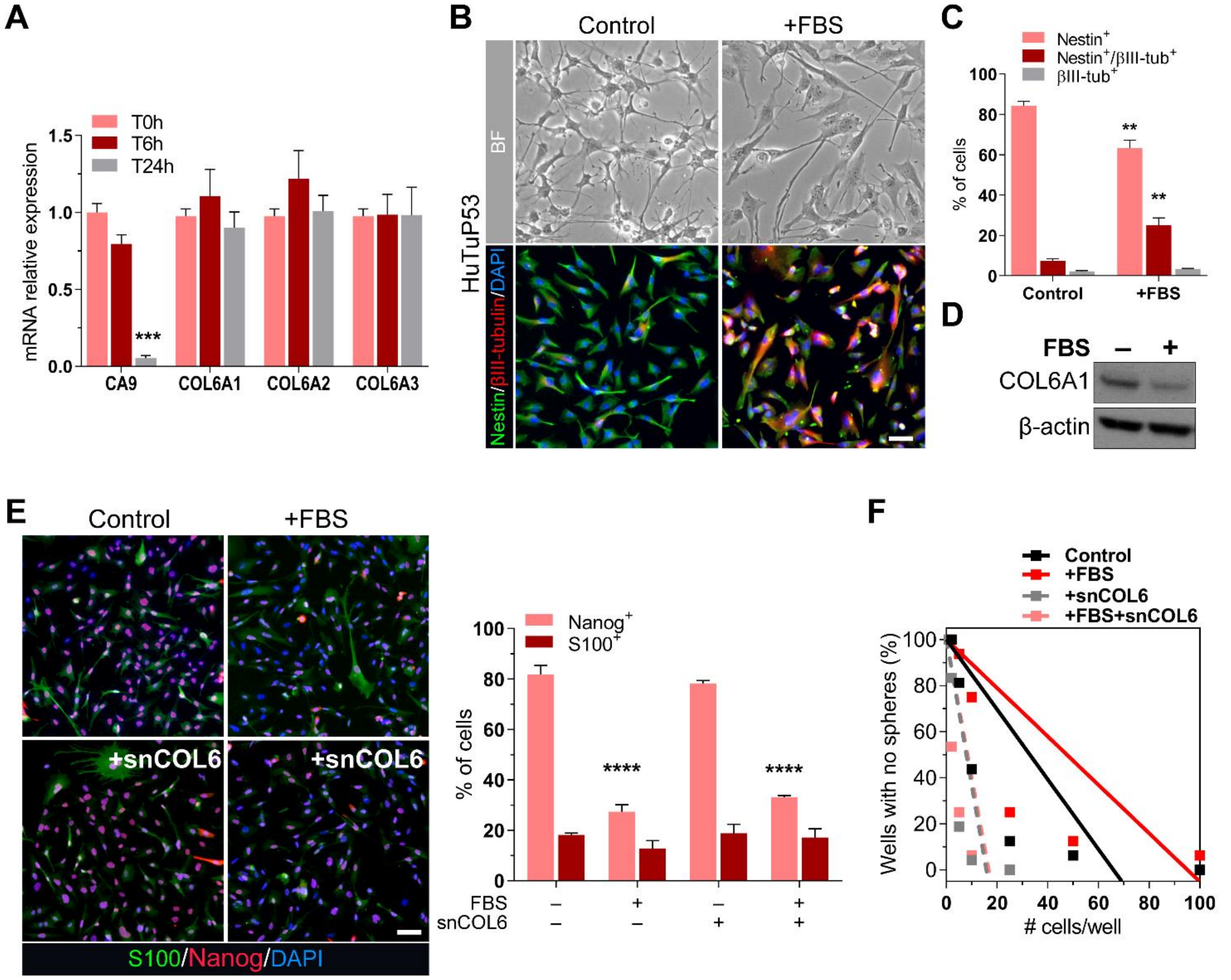

Supplementary Figure S4. COL6 expression does not depend on microenvironmental oxygen, but on GBM cell differentiation status. (**A**) Bar graph reporting the relative expression of the known hypoxia and HIF-1α target gene *CA9* and of *COL6A1*, *COL6A2* and *COL6A3* genes during time, when GBM cells (HuTuP53 and 82) are exposed to normoxic conditions (21% O_2). Statistical analysis by comparing oxygen exposed GBM cells with T0h through t test (n=4; ***, p <0.001). (**B**) Representative images displaying the morphological (top panels) and phenotypic (bottom) features of HuTuP53 GBM cells in undifferentiated conditions (control, left panels) and upon differentiation by 72 h exposure to 10% FBS-enriched medium. Top panels show bright field (BF) images, bottom panels display immunostaining for Nestin (green) and βIII-tubulin (red). Nuclei were counterstained with DAPI (blue). Original magnification: 10x; scale bar, 20 µm. (**C**) Relative quantification of Nestin^{+}/βIII-tubulin^{-}, Nestin^{+}/βIII-tubulin^{+} and Nestin^{-}/βIII-tubulin^{+} GBM cells in samples as in (**B**). Statistical analysis by comparing FBS treated cells with controls through t test (n=4; **, p <0.01). (**D**) Representative western blotting analysis for the α1(VI) COL6 chain (COL6A1) in HuTuP53 GBM cells in undifferentiated (– FBS) and differentiating (+ FBS) conditions. β-actin was used as loading control. (**E**) Representative immunofluorescence images displaying the combined expression of S100 (green) and Nanog (red) in GBM cells under undifferentiated (control) and differentiating (72 h FBS) conditions, in the absence of any further treatment (top) or in the presence of snCOL6 (1 µg/ml any other day; bottom). Nuclei were counterstained with DAPI (blue). Scale bar, 20 µm. On the right, relative quantification of immunofluorescence images (n=6). Statistical analysis was performed by comparing each experimental group with control cells (– FBS, – snCOL6) through One-way ANOVA with Dunnett's multiple comparisons test (****, p <0.0001). (**F**) Limiting dilution assay of HuTuP82 GBM cells in control conditions and upon 72 hr exposure to 10% FBS in combination or not with snCOL6 (1 µg/ml any other day).

Supplementary Figure S5

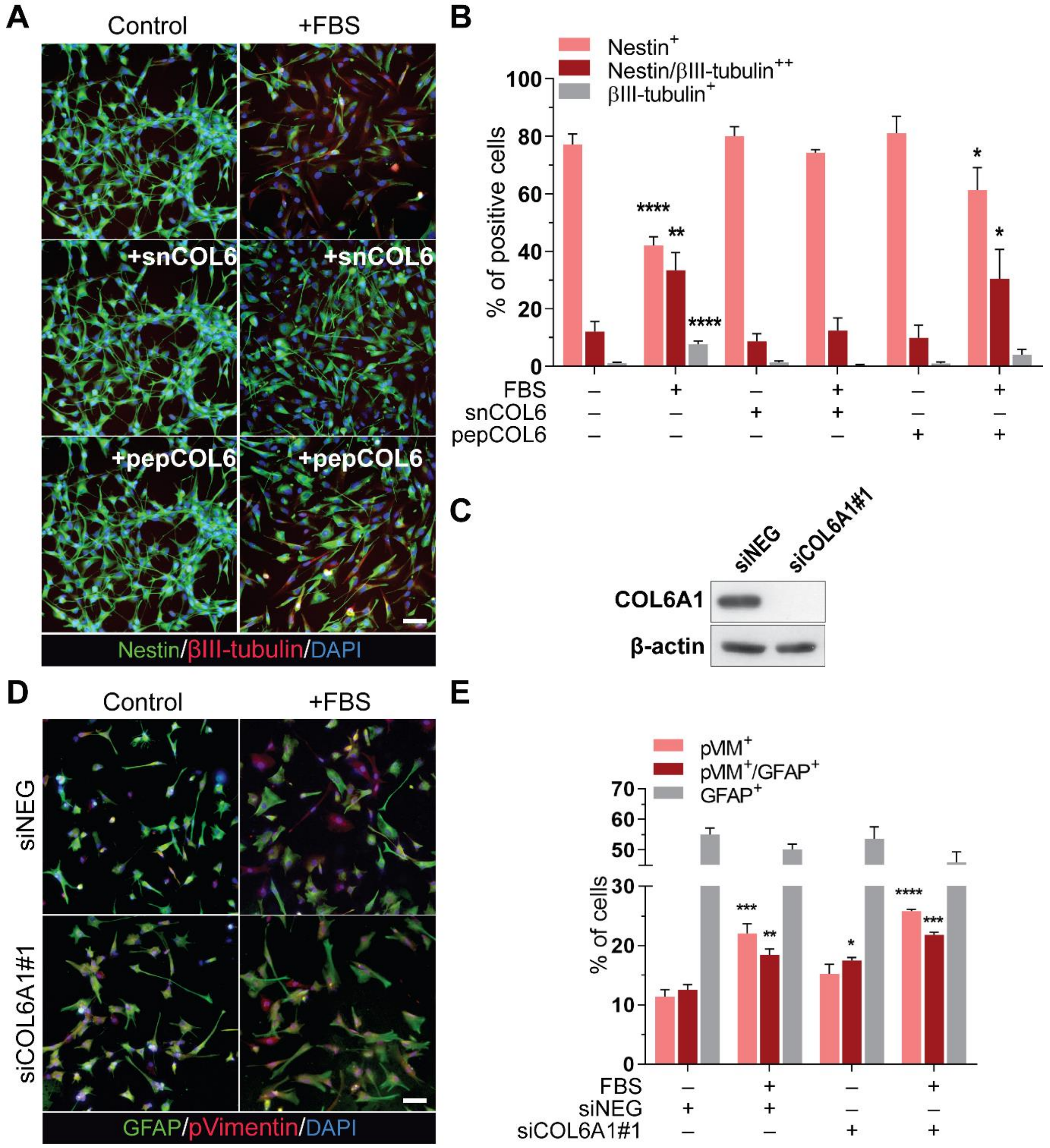

Supplementary Figure S5. Phenotypic assessment of GBM cell differentiation upon pepsinated COL6 treatment or COL6A1 silencing. (**A**, **B**) Representative immunofluorescence images (**A**) and relative quantification (**B**) of HuTuP82 GBM cells stained for Nestin (green) and βIII-tubulin (red) in control conditions (– FBS) and upon 72 hr exposure to 10% FBS, in the absence or presence of snCOL6 (1 µg/ml any other day) or pepCOL6 (250 ng/ml any other day) (*n*=8). Nuclei were counterstained with DAPI (blue). Original magnification: 10x; scale bar, 20 µm. Statistical analysis was performed by comparing each experimental group with control cells (– FBS, – COL6) through One-way ANOVA with Dunnett's multiple comparisons test (*, $p<0.05$; **, $p<0.01$; ****, $p<0.0001$). (**C**) Representative western blot analysis for the α1(VI) COL6 chain (COL6A1) in HuTuP82 GBM cells after 48h transfection with siNEG or siCOL6A1#1. β-actin was used as loading control. (**D**, **E**) Representative immunofluorescence images (**D**) and relative quantification (*n*=4) (**E**) of siNEG or siCOL6A1#1 transfected GBM cells (HuTuP82), treated as indicated and then stained for pVIM (red) and GFAP (green). Nuclei were counterstained with DAPI (blue). Original magnification: 10x; scale bar, 20 µm. Statistical analysis was performed by comparing each experimental group with siNEG control cells (– FBS) through One-way ANOVA with Dunnett's multiple comparisons test (*, $p<0.05$; **, $p<0.01$; ***, $p<0.001$; ****, $p<0.0001$).

Supplementary Figure S6

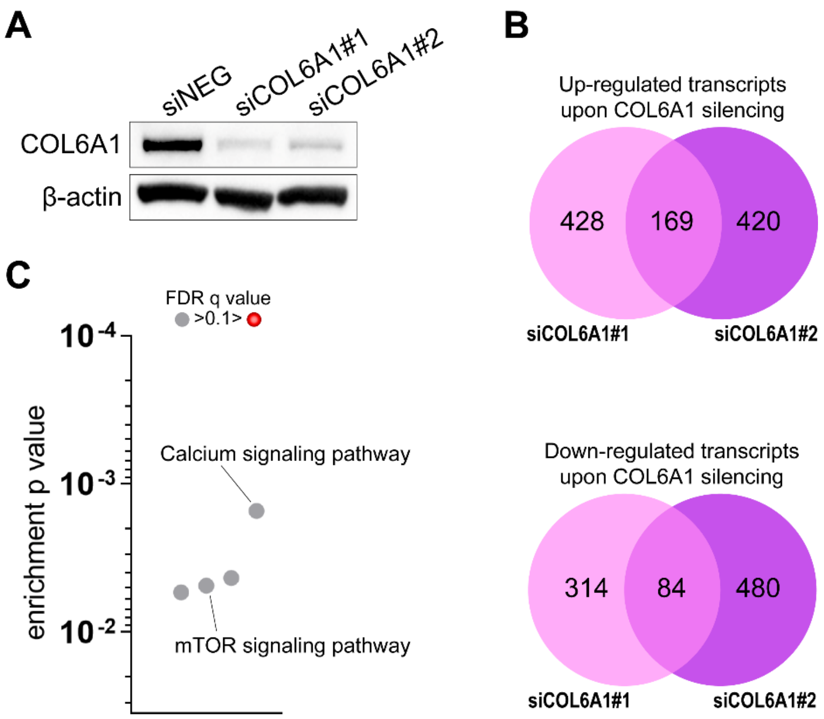

Supplementary Figure S6. COL6A1 silencing significantly affects GBM cell transcription. (**A**) Representative western blot analysis of the α1(VI) COL6 chain (COL6A1) in HuTuP82 cells transfected with siNEG or with two different siRNAs against COL6A1 (COL6A1#1 and #2). β-actin was used as loading control. (**B**) Venn diagrams identifying differentially expressed genes in COL6A1-silenced GBM cells, and upregulated (*n*=169; top) or down-regulated (*n*=84; bottom) by both siCOL6A1#1 and siCOL6A1#2. (**C**) Dot plot summarizing the positively enriched pathways in COL6-silenced HuTuP82 GBM cells by over-representation analysis of the C2cp gene sets.

Supplementary Figure S7

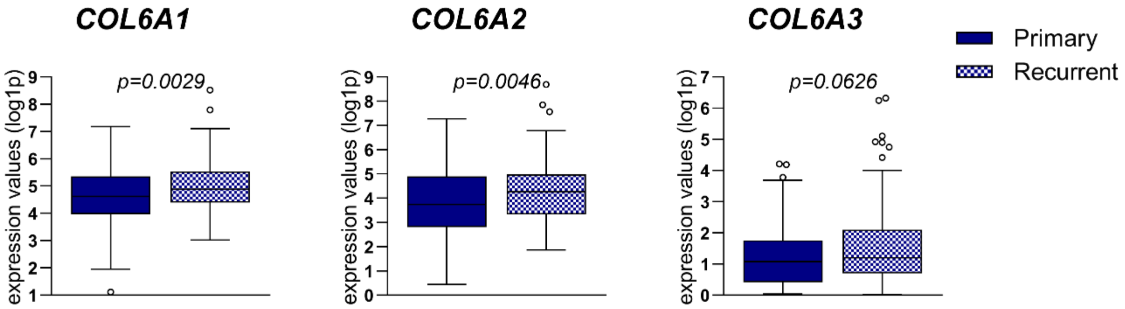

Supplementary Figure S7. Expression of COL6 genes in primary vs recurrent GBM tumors. Boxplots reporting the expression of *COL6A1*, *COL6A2* and *COL6A3* genes in primary (*n*=140) vs recurrent (*n*=109) GBM (WHO IV) samples from the CGGA patient dataset [5]. Statistical analysis by t test.

References

1. Bergamaschi A, Tagliabue E, Sorlie T, Naume B, Triulzi T, Orlandi R, et al. Extracellular matrix signature identifies breast cancer subgroups with different clinical outcome. J Pathol. 2008; 214: 357-67.

2. Sun L, Hui AM, Su Q, Vortmeyer A, Kotliarov Y, Pastorino S, et al. Neuronal and glioma-derived stem cell factor induces angiogenesis within the brain. Cancer Cell. 2006; 9: 287-300.

3. Murat A, Migliavacca E, Gorlia T, Lambiv WL, Shay T, Hamou MF, et al. Stem cell-related "self-renewal" signature and high epidermal growth factor receptor expression associated with resistance to concomitant chemoradiotherapy in glioblastoma. J Clin Oncol. 2008; 26: 3015-24.

4. Brennan CW, Verhaak RG, McKenna A, Campos B, Noushmehr H, Salama SR, et al. The somatic genomic landscape of glioblastoma. Cell. 2013; 155: 462-77.

5. Zhao Z, Zhang KN, Wang Q, Li G, Zeng F, Zhang Y, et al. Chinese Glioma Genome Atlas (CGGA): A Comprehensive Resource with Functional Genomic Data from Chinese Glioma Patients. Genomics Proteomics Bioinformatics. 2021; 19: 1-12.

6. Boso D, Rampazzo E, Zanon C, Bresolin S, Maule F, Porcu E, et al. HIF-1alpha/Wnt signaling-dependent control of gene transcription regulates neuronal differentiation of glioblastoma stem cells. Theranostics. 2019; 9: 4860-77.

7. Darmanis S, Sloan SA, Croote D, Mignardi M, Chernikova S, Samghababi P, et al. Single-Cell RNA-Seq Analysis of Infiltrating Neoplastic Cells at the Migrating Front of Human Glioblastoma. Cell Rep. 2017; 21: 1399-410.
